# Prognostic significance of the postoperative prognostic nutritional index in patients with glioblastoma: a retrospective study

**DOI:** 10.1186/s12885-021-08686-8

**Published:** 2021-08-21

**Authors:** Yoon Jung Kim, Hyongmin Oh, Sang Jin Lee, Kyung-Min Kim, Ho Kang, Chul-Kee Park, Hee-Pyoung Park

**Affiliations:** 1 Department of Anesthesiology and Pain Medicine, Seoul National University Hospital, Seoul National University College of Medicine, 101 Daehak-ro, Jongno-gu, Seoul, 03080 South Korea; 2 Department of Neurosurgery, Seoul National University Hospital, Seoul National University College of Medicine, 101 Daehak-ro, Jongno-gu, Seoul, 03080 South Korea

**Keywords:** Prognostic nutritional index, Glioblastoma, Overall survival, Surgery

## Abstract

**Background:**

The prognostic nutritional index (PNI) reflects immunonutritional status. We evaluated the effects of postoperative PNI and perioperative changes in the PNI on overall survival (OS) in glioblastoma (GBM) patients.

**Methods:**

Demographic, laboratory, and clinical data were retrospectively collected from 335 GBM patients. Preoperative and postoperative PNIs were calculated from serum albumin concentration and lymphocyte count, which were measured within 3 weeks before surgery and 1 month after surgery. Patients were classified into high (*n* = 206) or low (*n* = 129) postoperative PNI groups according to the postoperative PNI cutoff value and further classified into four groups according to the cutoff values of the preoperative and postoperative PNIs, as follows: Group HH (both high PNIs, *n* = 92), Group HL (high preoperative and low postoperative PNI, *n* = 70), Group LH (low preoperative and high postoperative PNI, *n* = 37), and Group LL (both low PNIs, *n* = 136).

**Results:**

The median OS was significantly longer in the high postoperative PNI (PNI ≥ 50.2) group than the low postoperative PNI (PNI < 50.2) group (24.0 vs. 15.0 months, *p* <  0.001). In multivariate analysis, high postoperative PNI was a significant predictor of OS. OS was significantly longer in Group HH than in Group LL and seemed longer in Group HH than in Group HL and in Group LH than in Group LL. OS was not different between Groups HH and LH or between Groups HL and LL.

**Conclusions:**

High postoperative PNI was associated with improved OS and perioperative changes in PNI may provide additional important information for prognostic prediction in GBM patients.

**Supplementary Information:**

The online version contains supplementary material available at 10.1186/s12885-021-08686-8.

## Background

Glioblastoma (GBM) is the most common malignant primary brain tumor characterized by high mortality and recurrence. Despite the use of Stupp protocol (postoperative radiotherapy plus concomitant and adjuvant temozolomide chemotherapy), the median overall survival (OS) is as short as 14.6 months [1, 2]. Moreover, there is a survival gap even among patients who are managed with the same treatment protocol. Numerous studies have attempted to identify prognostic factors in GBM patients. Well-documented prognostic parameters associated with a favorable outcome in patients with GBM include young age, high preoperative Karnofsky performance status (KPS) score, genetic composition (i.e., isocitrate dehydrogenase [IDH] mutation, O6-methylguanine-DNA methyltransferase [MGMT] promoter methylation), and more extensive surgical resection [3–15].

The prognostic nutritional index (PNI) is calculated from the serum level of albumin and lymphocyte count and reflects immunonutritional status [16]. Literature reviews have revealed the controversy about the role of preoperative PNI in predicting the prognosis of GBM patients [17–22]. However, no study has investigated the prognostic significance of the postoperative PNI in GBM patients. Moreover, the effects of combination of preoperative and postoperative PNIs on postoperative survival have not been studied in such patients. A few studies focusing on the prognostic role of postoperative PNI have reported that it is predictive of prognosis in patients with hepatocellular carcinoma and lung cancer and a high postoperative PNI is associated with a good prognosis in such patients [23, 24]. These studies indicate that the postoperative PNI rather than preoperative PNI can better reflect postoperative general conditions in patients undergoing hepatic and pulmonary tumor surgeries. The postoperative PNI may depend on postoperative clinical course and management. Previous studies demonstrated that postoperative complications had a negative relationship with the postoperative PNI [23, 25].

Therefore, we hypothesized that a high postoperative PNI would be associated with improved OS in GBM patients. This study evaluated the effects of the postoperative PNI and the change in perioperative PNI on OS in GBM patients and whether the postoperative PNI is a significant prognostic factor. We also identified demographic and clinical factors that contribute to the prognostic significance of postoperative PNI.

## Methods

This retrospective study was conducted after approval from the Institutional Review Board of Seoul National University Hospital (number: 2101–092-1189). The Institutional Review Board of Seoul National University Hospital waived the requirement for written informed consent because of the retrospective design of the study. We included patients newly diagnosed with histological GBM who underwent brain tumor surgery under general anesthesia (total intravenous anesthesia with propofol and continuous infusion of remifentanil) at Seoul National University Hospital from 2010 to 2016. Exclusion criteria were patients with surgical mortality, recurrent GBM, missing data on preoperative or postoperative laboratory examinations, and infectious or chronic autoimmune disease or glucocorticoid replacement, which can affect the immune system and nutritional status. In addition, patients with concurrent steroid use at the time of PNI calculations were excluded.

### Data collection

We retrospectively reviewed the electronic medical records of subjects to collect data categorized into four parts: preoperative data, including demographic information, comorbidities, daily activities, represented by the KPS score, preoperative laboratory findings (serum albumin concentration and lymphocyte count); intraoperative data, including surgical time and intraoperative transfusions; postoperative data, including postoperative laboratory findings (albumin levels and lymphocyte count), KPS score at hospital discharge, the extent of surgical resection, which was radiographically confirmed and classified into gross total resection, near-total resection, subtotal resection, partial resection, and biopsy, and postoperative adjuvant therapy (application and completion of the Stupp protocol [1], radiotherapy only, chemotherapy only, or none); and gene expression profiles, including MGMT methylation, epidermal growth factor receptor amplification, and the IDH mutation.

### Definition

The PNI was defined as 10 × albumin level (g/dL) + 0.005 × lymphocyte count (10^6^/L) [16], and the preoperative and postoperative PNIs were calculated from preoperative (within 3 weeks before surgery) and postoperative (1 month after surgery but before postoperative adjuvant therapy) laboratory findings, respectively. High and low PNIs were defined when a PNI value was greater than or equal to the optimal cutoff value of the PNI and less than the optimal cutoff value, respectively. OS was defined as the time interval from the date of surgery to the date of death, or the date of the last follow-up.

### Statistical analysis

The Kolmogorov-Smirnov test was used to evaluate the normality of the distributions of all continuous variables. The Student’s *t*-test was used to analyze normal variables, and the Mann-Whitney *U*-test was used to compare skewed variables. Categorical variables were analyzed using the chi-square or Fisher’s exact test. The OS of GBM patients was analyzed using the Kaplan-Meier method. Univariate analysis was first performed to identify predictive factors of postoperative survival in GBM patients. Variables with a *p*-value < 0.1 in univariate analysis and well-known significant factors of postoperative survival of GBM were entered into multivariate logistic regression analysis with the forward stepwise conditional method. Receiver operating characteristic curve analysis were performed to identify the optimal cutoff values of the preoperative and postoperative PNIs to predict OS. The optimal cut-off point was determined by maximizing the sum of sensitivity and specificity (Youden’s index). To evaluate the effects of a change in the perioperative PNI on OS, patients were reclassified into four groups according to the cutoff values of the preoperative and postoperative PNIs: Group HH (high preoperative PNI + high postoperative PNI group), Group HL (high preoperative PNI + low postoperative PNI group), Group LH (low preoperative PNI + high postoperative PNI group), and Group LL (low preoperative PNI + low postoperative PNI group). Differences in OS among the four groups were analyzed using the Kaplan-Meier method and the log-rank test. The alpha value was adjusted with the Bonferroni correction to compensate for multiple comparisons, and the statistical significance of the alpha value was < 0.008 (0.05/6). The relationships between preoperative and postoperative PNIs and other clinical characteristics were evaluated with the Pearson correlation test.

All statistical analyses were conducted using SPSS statistical software for Windows, version 25.0 (IBM, Armonk, NY, USA) and R version 4.0.3 (The R Foundation for Statistical Computing, Vienna, Austria). A *p*-value < 0.05 was considered significant.

## Results

During the entire study period, 495 patients were diagnosed with histologically confirmed GBM. Among them, 160 patients (95 underwent surgery under local anesthesia, two died, 44 were lost to follow-up due to transfer to other local hospitals for postoperative adjuvant therapy, and 19 had incomplete laboratory data related to the PNI) were excluded from the study. Finally, 335 patients were included in the data analysis.

The preoperative and postoperative PNIs revealed area under the curve values of 0.579 (95% CI; 0.496–0.661; *P* = 0.061) and 0.599 (0.522–0.676; *P* = 0.018) for OS, respectively. The optimal cut-off values for the preoperative and postoperative PNIs were 50.1 and 50.2, respectively.

Patients in the high preoperative PNI group were younger than those in the low preoperative PNI group, and the high preoperative PNI group had a greater proportion of male patients and patients with preoperative and postoperative KPS score ≥ 70 than the low preoperative PNI group. (Table 1). Patients were younger, more patients had preoperative and postoperative KPS scores ≥70, underwent more frequent gross total resection of GBM and completion of Stupp protocol in the high postoperative PNI group.

**Table 1:** Demographic, perioperative laboratory results, genetic, and postoperative treatment-related data

	Preoperative PNI			Postoperative PNI		
	Low (PNI < 50. 1) (*n* = 173)	High (PNI ≥ 50. 1) (*n* = 162)	*P* value	Low (PNI < 50. 2) (*n* = 206)	High (PNI ≥ 50. 2) (*n* = 129)	*P* value
Age (yr)	56.5 ± 13.9	52.3 ± 13.7	0.005	57.2 ± 13.9	50.1 ± 12.9	< 0.001
< 60	96 (55.5%)	108 (66.7%)	0.047	108 (52.4%)	96 (74.4%)	< 0.001
< 70	136 (78.6%)	148 (91.4%)	0.002	161 (78.2%)	123 (95.3%)	< 0.001
Male gender (n)	91 (52.6%)	104 (64.2%)	0.041	114 (55.3%)	81 (62.8%)	0.218
BMI (kg/m^2^)	22.6 ± 3.2	23.5 ± 3.2	0.019	22.7 ± 3.2	23.6 ± 3.1	0.021
ASA physical status (n)			0.116			0.235
I	57 (32.9%)	63 (38.9%)		69 (33.5%)	51 (39.5%)	
II	93 (53.8%)	88 (54.3%)		112 (54.4%)	69 (53.5%)	
III	23 (13.3%)	11 (6.8%)		25 (12.1%)	9 (7.0%)	
Comorbidity (n)						
Hypertension	45 (26.0%)	46 (28.4%)	0.713	58 (28.2%)	33 (25.6%)	0.697
Diabetes mellitus	16 (9.2%)	9 (5.6%)	0.281	16 (7.8%)	9 (7.0%)	0.957
Cardiac disease	13 (7.5%)	6 (3.7%)	0.204	16 (7.8%)	3 (2.3%)	0.050
Respiratory disease	7 (4.0%)	4 (2.5%)	0.544	11 (5.3%)	0 (0.0%)	0.008
Liver disease	9 (5.2%)	8 (4.9%)	1.000	11 (5.3%)	6 (4.7%)	0.981
Renal disease	3 (1.7%)	0 (0.0%)	0.249	3 (1.5%)	0 (0.0%)	0.287
Cerebrovascular disease	12 (6.9%)	6 (3.7%)	0.285	10 (4.9%)	8 (6.2%)	0.777
Extracranial malignancy	11 (6.4%)	4 (2.5%)	0.113	12 (5.8%)	3 (2.3%)	0.177
Daily activity						
Preoperative KPS score	80 (70–90)	90 (80–90)	0.009	85 (70–90)	90 (80–90)	0.023
≥ 70 (n)	144 (83.2%)	154 (95.1%)	0.001	177 (85.9%)	121 (93.8%)	0.040
KPS score at hospital discharge	90 (70–90)	90 (80–90)	0.024	90 (70–90)	90 (80–90)	< 0.001
≥ 70 (n)	145 (83.8%)	146 (90.1%)	0.122	166 (80.6%)	125 (96.9%)	< 0.001
Laboratory findings						
Albumin (g/dL)						
Preoperative	3.9 ± 0.4	4.4 ± 0.3	< 0.001	4.1 ± 0.4	4.3 ± 0.4	< 0.001
Postoperative	3.9 ± 0.4	4.1 ± 0.3	< 0.001	3.8 ± 0.4	4.3 ± 0.2	< 0.001
Delta (preoperative – postoperative)	−0.01 ± 0.49	−0.26 ± 0.38	< 0.001	−0.23 ± 0.49	0.03 ± 0.35	< 0.001
Lymphocyte count (10^6^/L)						
Preoperative	1280.8 ± 495.2	2064.3 ± 667.6	< 0.001	1521.9 ± 646.7	1879.6 ± 736.3	< 0.001
< 1500 (n)	127 (73.4%)	37 (22.8%)	< 0.001	116 (56.3%)	48 (37.2%)	0.001
Postoperative	1558.5 ± 646.7	1827.0 ± 578.6	< 0.001	1422.4 ± 466.5	2113.1 ± 621.4	< 0.001
< 1500 (n)	89 (51.4%)	47 (29.0%)	< 0.001	120 (58.3%)	16 (12.4%)	< 0.001
Delta (preoperative – postoperative)	− 277.8 ± 680.1	237.3 ± 755.2	< 0.001	99.5 ± 702.4	−233.4 ± 808.5	< 0.001
PNI						
Preoperative	45.5 ± 3.6	54.4 ± 3.2	< 0.001	48.2 ± 5.2	52.4 ± 5.3	< 0.001
Postoperative	46.8 ± 5.2	50.6 ± 5.1	< 0.001	45.4 ± 4.0	53.8 ± 3.0	< 0.001
Delta (preoperative – postoperative)	−1.3 ± 5.8	3.8 ± 5.2	< 0.001	2.8 ± 5.9	−1.4 ± 5.3	< 0.001
Surgery time (hr)	4.4 ± 1.3	4.5 ± 1.4	0.581	4.4 ± 1.4	4.5 ± 1.2	0.471
Intraoperative transfusion (n)	41 (23.7%)	25 (15.4%)	0.077	54 (26.2%)	12 (9.3%)	< 0.001
Tumor resection (n)			0.642			0.072
Gross total	102 (59.0%)	107 (66.0%)	0.220	119 (57.8%)	90 (69.8%)	0.037
Near total	24 (13.9%)	16 (9.9%)	0.338	24 (11.7%)	16 (12.4%)	0.973
Subtotal	32 (18.5%)	28 (17.3%)	0.883	42 (20.4%)	18 (14.0%)	0.178
Partial	8 (4.6%)	7 (4.3%)	1.000	11 (5.3%)	4 (3.1%)	0.422
Biopsy	7 (4.0%)	4 (2.5%)	0.544	10 (4.9%)	1 (0.8%)	0.056
Gene expression profiles (n)						
MGMT Promoter methylation*	97 (56.4%)	80 (50.0%)	0.291	110 (54.2%)	67 (51.9%)	0.774
EGFR amplification^†^	45 (26.0%)	41 (25.6%)	1.000	53 (25.9%)	33 (25.8%)	1.000
IDH mutation^‡^	19 (12.2%)	30 (19.6%)	0.103	27 (14.4%)	22 (18.0%)	0.493
Postoperative new neurologic deficit (n)	43 (24.9%)	24 (14.8%)	0.031	54 (26.2%)	13 (10.1%)	< 0.001
Postoperative treatment (n)			0.033			0.030
Stupp protocol	151 (87.3%)	151 (93.2%)	0.102	180 (87.4%)	122 (94.6%)	0.050
Completed	99 (57.2%)	104 (64.2%)	0.219	113 (54.9%)	90 (69.8%)	0.009
Chemotherapy	6 (3.5%)	5 (3.1%)	1.000	7 (3.4%)	4 (3.1%)	1.000
Radiotherapy	1 (0.6%)	3 (1.9%)	0.357	2 (1.0%)	2 (1.6%)	0.641
None	15 (8.7%)	3 (1.9%)	0.007	17 (8.3%)	1 (0.8%)	0.002

Data are expressed as number (proportion), mean ± standard deviation, or median (interquartile range). *BMI* body mass index, *ASA* American Society of Anesthesiologists, *KPS* Karnofsky performance status, delta; preoperative –postoperative, *PNI* prognostic nutritional index, *MGMT* O6-methylguanine-DNA methyltransferase, *EGFR* epidermal growth factor receptor, *IDH* isocitrate dehydrogenase. Data are expressed as number (proportion), mean ± standard deviation, or median (interquartile range). *: *n* = 172 in preoperative PNI low group, *n* = 160 in preoperative PNI high group, and *n* = 203 in postoperative PNI low group. †: *n =* 160 in preoperative PNI high group, and *n* = 205 in postoperative PNI low group, and *n* = 128 in postoperative PNI high group. ^‡^: *n* = 156 in preoperative PNI low group, *n* = 153 in preoperative PNI high group, and *n* = 187 in postoperative PNI low group, and *n* = 122 in postoperative PNI high group

**Fig. 1:**
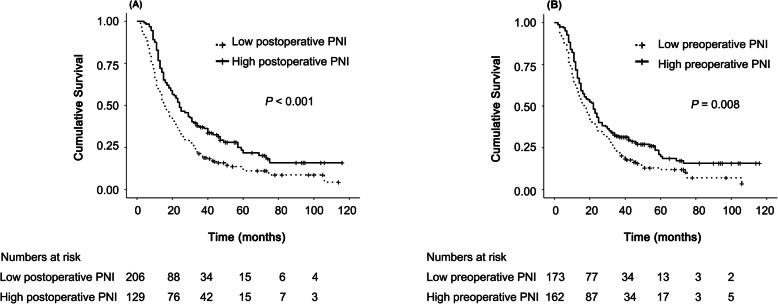
Kaplan-Meier curves for cumulative survival according to (**A**) postoperative PNI and (**B**) preoperative PNI. PNI: prognostic nutrition index

A total of 233 (69.6%), 92 (27.5%), and 66 (19.7%) patients survived 1, 3, and 5 years after surgery, respectively. Fifty-seven (17.0%) patients survived at the last follow-up. The median (95% CI) OS of all patients was 19.0 (15.9–22.1) months. The duration of OS was significantly longer in the high postoperative PNI group than in the low postoperative PNI group (median OS: 24.0 vs. 15.0 months, *P* <  0.001, Fig. 1A) and longer in the high preoperative PNI group than in the low preoperative PNI group (median OS: 22.0 vs. 17.0 months, *P* = 0.008, Fig. 1B).

Significant predictive factors for postoperative OS in multivariate analysis (Table 2) were age <  60 years (odd ratio [95% CI], 2.07 [1.02–4.21]; *P* = 0.045), high postoperative PNI (2.17 [1.14–4.10]; *P* = 0.018), MGMT promoter methylation (2.61 [1.33–5.11]; *P* = 0.005), and completion of the Stupp protocol (5.39 [2.18–13.27]; *P* <  0.001).

**Table 2:** Univariate and multivariate analysis for predictive factors associated with postoperative overall survival in glioblastoma patients

	Univariate analysis			Multivariate analysis*		
	Odds ratio	95% CI	*P* value	Odds ratio	95% CI	*P* value
Age < 60 years (n)	2.50	1.29–4.84	0.007	2.07	1.02–4.21	0.045
Male gender (n)	0.59	0.33–1.05	0.070			
Preoperative KPS score ≥ 70 (n)	1.07	0.42–2.69	0.891			
KPS score at hospital discharge ≥70 (n)	3.11	0.93–10.43	0.066			
Albumin (g/dL)						
Preoperative	1.93	0.93–4.01	0.078			
Postoperative	3.43	1.47–7.98	0.004			
High preoperative PNI (PNI ≥ 50. 1) (n)	2.26	1.25–4.10	0.007			
High postoperative PNI (PNI ≥ 50. 2) (n)	2.39	1.34–4.26	0.003	2.17	1.14–4.10	0.018
Extent of surgical resection			0.083			
Gross total resection	Reference					
Near total resection	0.09	0.01–0.70	0.021			
Subtotal resection	0.64	0.39–1.40	0.268			
Partial resection	0.26	0.03–2.03	0.199			
Biopsy	0.36	0.05–2.92	0.342			
Postoperative new neurologic deficit (n)	0.61	0.27–1.35	0.220			
Gene expression profiles (n)						
MGMT Promoter methylation	2.62	1.40–4.89	0.003	2.61	1.33–5.11	0.005
IDH mutation	2.08	1.03–4.19	0.041			
Completed Stupp protocol (n)	5.84	2.56–13.32	< 0.001	5.39	2.18–13.27	< 0.001

*KPS* Karnofsky performance status, *PNI* prognostic nutritional index, *MGMT* O6-methylguanine-DNA methyltransferase, *IDH* isocitrate dehydrogenase, *RT* radiation therapy. *Multivariate analysis with the forward stepwise conditional method was performed and male gender, preoperative KPS score ≥ 70, KPS score at hospital discharge ≥70, preoperative and postoperative serum albumin level, high preoperative PNI, the extent of surgical resection, postoperative new neurologic deficit, IDH mutation, were adjusted. Nagelkerke R^2^ statistic in step 4 is 0.208

**Fig. 2:**
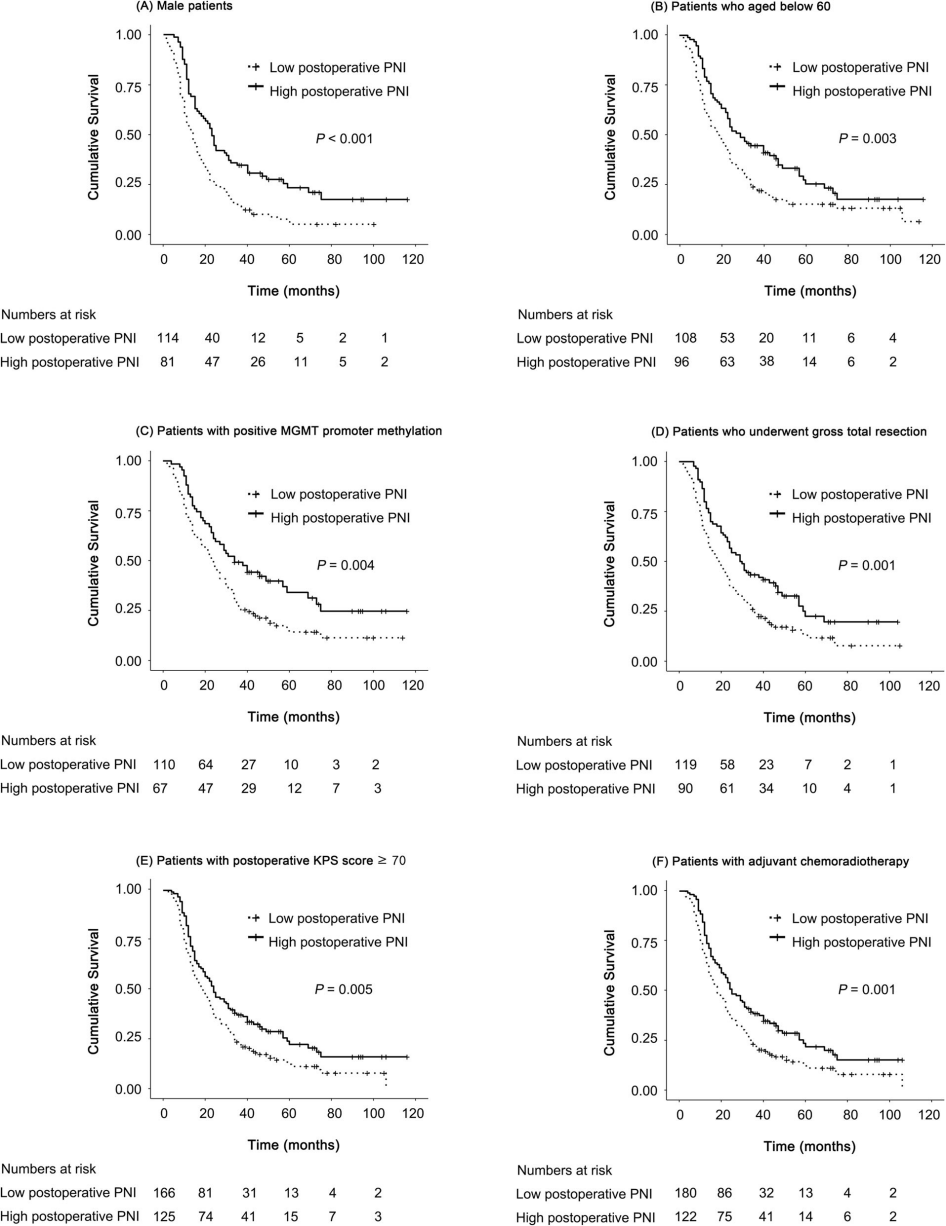
Kaplan-Meier curves for cumulative survival in subgroups. (**A**) male patients, (**B**) subjects who aged below 60, (**C**) patients with positive MGMT promoter methylation, (**D**) patients who underwent gross total resection, (**E**) patients with postoperative KPS score ≥ 70, and (**F**) patients with adjuvant chemoradiotherapy. PNI: prognostic nutrition index; MGMT: O6-methylguanine-DNA methyltransferase; KPS: Karnofsky performance status

In subgroup analysis, OS was significantly longer in the high postoperative PNI group than in the low postoperative PNI group in patients who were male (*P <*  0.001) and young (age < 60 years, *P* = 0.003), had MGMT promoter methylation (*P* = 0.004), received gross total resection of GBM (*P* = 0.001), had a postoperative KPS score ≥ 70 (*P* = 0.005), and received postoperative chemoradiotherapy, *P =* 0.001, Fig. 2A-F). OS was significantly longer in the high preoperative PNI group than in the low preoperative PNI group in patients who were male and young, had an unmethylated MGMT promoter, underwent gross total resection, had a preoperative KPS score ≥ 70 and postoperative KPS score <  70, and received adjuvant chemoradiotherapy (Supplementary Fig. 1).

**Fig. 3:**
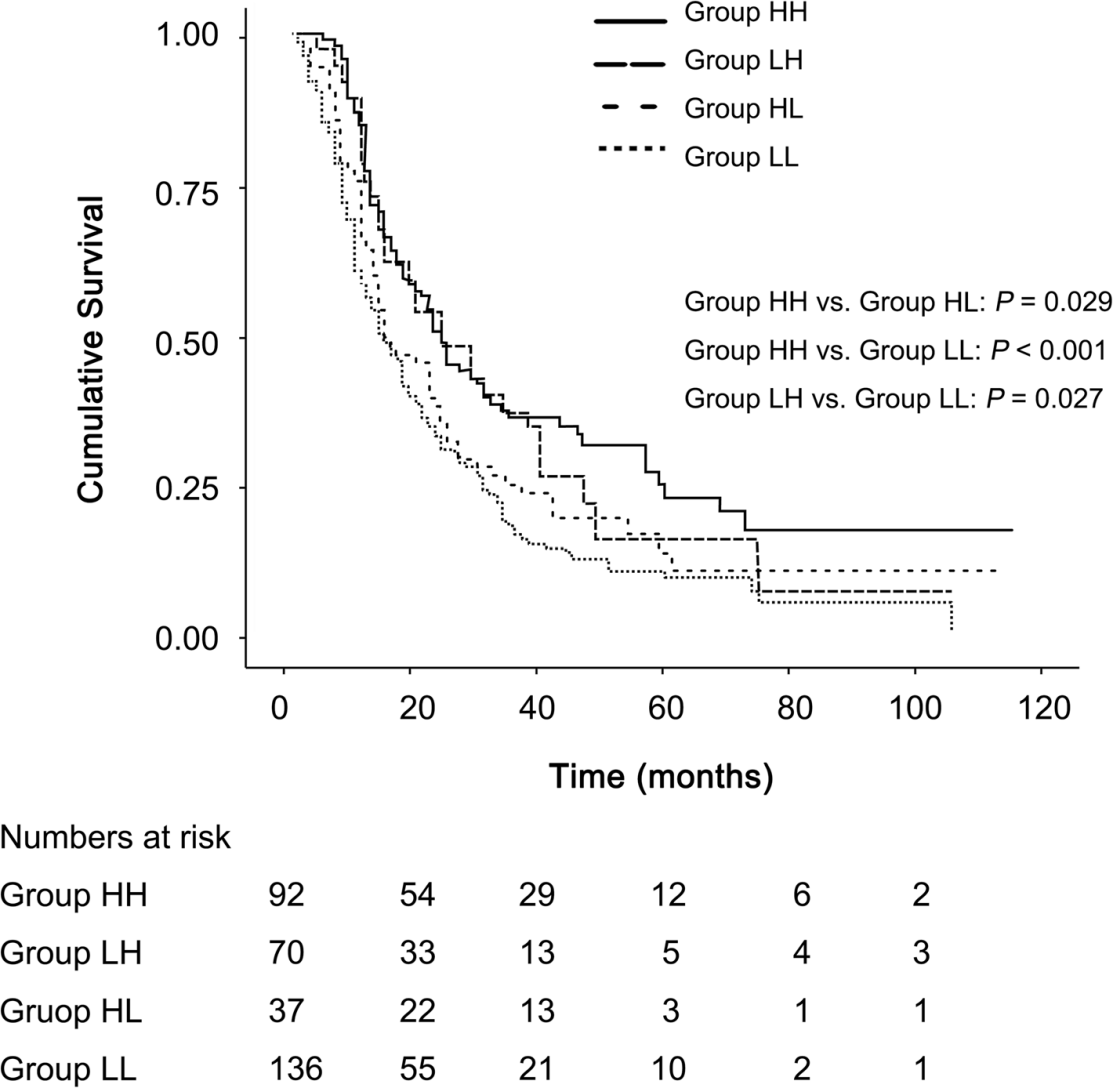
Kaplan-Meier curves for cumulative survival according to perioperative changes of PNI. PNI: prognostic nutrition index; Group HH: high preoperative PNI and high postoperative PNI (solid line); Group LH: low preoperative PNI and high postoperative PNI (long dashed line); Group HL; high preoperative PNI and low postoperative PNI (short dashed line); Group LL: low preoperative PNI and low postoperative PNI (dotted line). Because Bonferroni correction of the alpha value was performed to compensate multiple comparisons, a *p-*value < 0.008 (0.05/6) was considered statistically significant

A significant difference in OS was observed among the four groups (*P* = 0.001, Fig. 3). The median (95% CI) OS durations were 24.0 (18.9–29.1), 15.0 (9.7–20.3), 24.0 (14.1–33.9), and 15.0 (11.2–18.4) months in Group HH (*n* = 92), Group HL (*n* = 70), Group LH (*n* = 37), and Group LL (*n* = 136), respectively. A significant difference in OS was observed between Group HH and Group LL (*P* <  0.001). OS was longer in Group HH than in Group HL (*P* = 0.029), and in Group LH than in Group LL (*P* = 0.027). OS was not significantly different between Groups HH and LH or between Groups HL and LL.

Both preoperative and postoperative PNIs showed significant negative correlation with age (*r =* − 0.214 and − 0.321, *P* <  0.001 in both). Preoperative PNI was positively correlated with preoperative and postoperative KPS scores (*r =* 0.221 and 0.147, *P <*  0.001 and *P* = 0.007 respectively) but was not correlated with completion of the Stupp protocol. Postoperative PNI was positively correlated with preoperative and postoperative KPS scores and completion of the Stupp protocol (*r =* 0.231, 0.442, and 0.215, *P <*  0.001 respectively). The delta PNI (preoperative PNI – postoperative PNI) was negatively correlated with the duration of OS in patients with mortality (*r =* − 0.160, *P =* 0.007). Also, the delta PNI was higher in patients with 1-year and 3-year mortalities, compared with those with 1-year and 3-year survivals (1-year: 2.2 ± 6.2 vs. 0.7 ± 5.9, *P* = 0.029; 3-year: 1.6 ± 6.0 vs. 0.1 ± 6.0, *P* = 0.040).

## Discussion

Despite recent advancements in diagnostic and therapeutic techniques, GBM is associated with poor clinical outcomes. Thus, it is relevant to identify potential prognostic serum biomarkers for poor clinical outcomes in GBM patients to help stratify patients. The PNI, which is easily calculated from laboratory tests, is a simple, economic, and convenient parameter for that purpose. This is the first study to evaluate the effects of postoperative PNI and perioperative changes in the PNI on prognosis in patients with GBM. This study demonstrated that high postoperative PNI was a significant independent predictor of OS in GBM patients and the change in the perioperative PNI helped predict the postoperative prognosis.

In clinical practice, various nutritional indices incorporating serum albumin concentration have been used to reflect nutritional status including PNI, the albumin/globulin ratio, and nutritional risk index [20, 23, 24, 26, 27]. PNI is an immunonutritional indicator. A recent study demonstrated that nutritional status was associated with postoperative survival in patients with GBM, and PNI rather than other nutritional indices robustly reflected the nutritional status [22]. However, All previous studies investigating the relationship between the PNI and prognosis of GBM focused only on the effect of preoperative PNI on OS and reported inconsistent results [17–22]. In contrast with previous studies, we evaluated the prognostic significance of the postoperative PNI in GBM patients and found a positive association between postoperative PNI and OS. Since GBM is characterized by rapid progression, quick and complete surgical resection is the treatment of choice. In our clinic, when a patient was suspected of GBM, the surgery was performed as soon as possible. It took an average of 7 days, up to 10 days, from the visit of the suspected patient to the surgery. Therefore, intensive nutritional intervention was practically available in the postoperative period in patients with GBM. Moreover, we think that postoperative PNI can better reflect postoperative general conditions than preoperative PNI and that improving nutritional status just before starting postoperative adjuvant therapy (for example, the Stupp protocol) can be helpful in achieving a favorable outcome. For the reason, we chose postoperative PNI and investigated its association with prognosis in patients with GBM.

In the present study, some results showed that the postoperative PNI might take an advantage over the preoperative PNI in predicting prognosis in GBM patients. First, a high postoperative PNI was an independent prognostic factor for GBM in multivariate analysis, whereas a high preoperative PNI was not. Second, the high postoperative PNI was related to increased survival in the subgroups of MGMT promoter methylation and postoperative KPS score ≥ 70, while the high preoperative PNI increased survival in the subgroups of MGMT promoter unmethylation and postoperative KPS score <  70. It is well known that MGMT promoter methylation and high KPS score were associated with favorable outcomes in patients with GBM [3, 8, 10, 12, 13]. Third, a significant difference in OS was observed among the four groups according to perioperative changes in the PNI. In other words, patients in Group HH (high preoperative and high postoperative PNI) showed significantly better OS than those in Group LL. OS was likely to be better in Group HH than Group HL, and in Group LH than in Group LL. However, OS was not significantly different between Groups HH and LH or between Groups HL and LL. Similar to our results, previous retrospective studies have demonstrated that the combination of the preoperative and postoperative PNI played an important role in predicting the precise postoperative prognosis in patients with various malignant tumors [25, 27, 28]. Moreover, the delta PNI showed a significant negative correlation with the duration of OS in patients with mortality. Namely, it was significantly higher in patients with 1-year and 3-year mortalities than those without respectively. Finally, completion of the Stupp protocol was positively correlated with postoperative PNI, not preoperative PNI. In this study, completion of the Stupp protocol was the most powerful predictor of favorable outcomes in patients with GBM. Similarly, previous studies have been highlighted the importance of completion of the Stupp protocol in predicting a favorable prognosis in GBM patients [1, 2]. Taken together, our results suggest that although both PNIs played a key role in predicting OS in GBM patients, the postoperative PNI rather than the preoperative PNI may provide more useful information for predicting GBM prognosis.

An important component of the PNI is the serum level of albumin, one of the simplest and most studied factors representing nutritional status. The postoperative serum level of albumin was associated with OS in GBM patients in univariate analysis, but it was not a predictor in multivariate analysis. Previous studies investigating the association between preoperative serum albumin concentration and prognosis of GBM reported that low serum albumin concentration was an independent poor prognostic factor [6, 29, 30]. Lymphocytes, which are the other component of the PNI, play a key role in cell-mediated immunity. Cell-mediated immunosuppression is well documented in GBM patients [31, 32]. GBM itself affects the number and function of T-lymphocytes. Cancer-induced chronic inflammation is associated with increased tumor proliferation and metastasis, and also has an immunosuppressive effect with a reduced lymphocyte count and impaired lymphocyte function [33, 34]. Hematopoiesis of lymphocytes can also be affected by nutritional status and anti-cancer treatment. Their production in bone marrow significantly decreases in patients who are protein malnourished [35, 36]. Postoperative adjuvant radiotherapy and chemotherapy inhibit proliferation of hematopoietic progenitor cells and block differentiation into lymphocytes, leading to lymphopenia. A previous clinical investigation reported a close relationship between postoperative treatment-related lymphopenia and a poor prognosis of GBM in elderly patients [37]. Taken together, these findings suggest that perioperative interventions, such as aggressive nutritional support before surgery and during the early postoperative period, may improve the prognosis of GBM patients by increasing both of the PNIs.

In this study, the postoperative PNI was calculated from serum albumin concentration and lymphocyte count, which were measured at 1 month postoperatively but before the commencement of postoperative adjuvant therapy. The postoperative PNI reflected the immune-nutritional status of the patients who had recovered from the surgery and were ready for postoperative adjuvant therapy (chemotherapy and/or radiotherapy). Our results showed that postoperative PNI was positively correlated with postoperative KPS score and completion of the Stupp protocol. GBM patients with poor nutritional status can have poor functional status and decreased functional reserves to endure various postoperative surgical stress responses. Also, they can be vulnerable to the adverse effects of postoperative adjuvant therapy, resulting in poor tolerance to adjuvant therapy and subsequent poor clinical outcomes. Although the Stupp protocol increases survival time, the standard Stupp protocol might be too aggressive to be tolerable for malnourished GBM patients. In this study, the incidence of completed Stupp protocol was significantly lower in patients with low postoperative PNI. In clinical practice, when deciding on individualized postoperative treatment options for patients with GBM, various factors such as perioperative functional status, patient age, immunonutritional status, and tolerability to the treatment should be considered [38].

Our study had some potential limitations. First, as this was a non-randomized retrospective study, there was a possibility of unexpected selection bias. In addition, the data were collected from a single institution. Second, low Nagelkerke R^2^ values in multivariate analysis to predict postoperative survival suggest that clinically valid prognostic parameters may have been omitted. Also, the discrimination power of postoperative PNI as a single predictive factor was poor. However, combined analysis of preoperative and postoperative PNIs helped stratify patients by providing the precise prognosis for patients with GBM. Third, patients who underwent a GBM biopsy under local anesthesia were excluded from the data analysis because anesthetics can affect OS and disease-free survival of various cancers [39, 40]. Therefore, caution is needed when interpreting our results.

## Conclusions

In conclusion, a high postoperative PNI was associated with improved postoperative OS in GBM patients. Combined analysis of preoperative and postoperative PNIs may provide additional supportive information on postoperative prognosis in such patients. A further large-scaled prospective study is needed to confirm our results and determine whether perioperative interventions to increase both PNIs would improve the prognosis of GBM patients.

## Supplementary Information

**Additional file 1: Supplementary Fig. 1.** Kaplan-Meier curves for cumulative survival in (A) male patients, (B) subjects who aged below 60, (C) patients with negative MGMT promoter methylation, (D) patients who underwent gross total resection, (E) patients with preoperative KPS score ≥ 70, (F) patients with adjuvant chemoradiotherapy, and (G) patients with postoperative KPS score < 70. PNI: prognostic nutrition index; MGMT: O6-methylguanine-DNA methyltransferase; KPS: Karnofsky performance status.

## Data Availability

The datasets generated during and/or analyzed during the current study are available from the corresponding author on reasonable request.

## Abbreviations

GBM: Glioblastoma
KPS: Karnofsky performance status
OS: Overall survival
MGMT: O6-methylguanine-DNA methyltransferase
PNI: Prognostic nutritional index
IDH: Isocitrate dehydrogenase
IRB: Institutional Review Board
Group HH: High preoperative PNI + high postoperative PNI group
Group HL: High preoperative PNI + low postoperative PNI group
Group LH: Low preoperative PNI + high postoperative PNI group
Group LL: Low preoperative PNI + low postoperative PNI group

## References

[1] Stupp R, Mason WP, van den Bent MJ, Weller M, Fisher B, Taphoorn MJB, Belanger K, Brandes AA, Marosi C, Bogdahn U, Curschmann J, Janzer RC, Ludwin SK, Gorlia T, Allgeier A, Lacombe D, Cairncross JG, Eisenhauer E, Mirimanoff RO (2005). Radiotherapy plus concomitant and adjuvant Temozolomide for glioblastoma. N Engl J Med.

[2] Stupp R, Hegi ME, Mason WP, van den Bent MJ, Taphoorn MJB, Janzer RC, Ludwin SK, Allgeier A, Fisher B, Belanger K, Hau P, Brandes AA, Gijtenbeek J, Marosi C, Vecht CJ, Mokhtari K, Wesseling P, Villa S, Eisenhauer E, Gorlia T, Weller M, Lacombe D, Cairncross JG, Mirimanoff RO, European Organisation for Research and Treatment of Cancer Brain Tumour and Radiation Oncology Groups, National Cancer Institute of Canada Clinical Trials Group (2009). Effects of radiotherapy with concomitant and adjuvant temozolomide versus radiotherapy alone on survival in glioblastoma in a randomised phase III study: 5-year analysis of the EORTC-NCIC trial. Lancet Oncol.

[3] Binabaj MM, Bahrami A, Shahidsales S, Joodi M, Joudi Mashhad M, Hassanian SM, Anvari K, Avan A (2018). The prognostic value of MGMT promoter methylation in glioblastoma: a meta-analysis of clinical trials. J Cell Physiol.

[4] Songtao Q, Lei Y, Si G, Yanqing D, Huixia H, Xuelin Z, Lanxiao W, Fei Y (2012). IDH mutations predict longer survival and response to temozolomide in secondary glioblastoma. Cancer Sci.

[5] Smith JS, Tachibana I, Passe SM, Huntley BK, Borell TJ, Iturria N, O'Fallon JR, Schaefer PL, Scheithauer BW, James CD, Buckner JC, Jenkins RB (2001). PTEN mutation, EGFR amplification, and outcome in patients with anaplastic astrocytoma and glioblastoma Multiforme. JNCI.

[6] Han S, Huang Y, Li Z, Hou H, Wu A (2015). The prognostic role of preoperative serum albumin levels in glioblastoma patients. BMC Cancer.

[7] Gupta D, Lis CG (2010). Pretreatment serum albumin as a predictor of cancer survival: a systematic review of the epidemiological literature. Nutr J.

[8] Gorlia T, van den Bent MJ, Hegi ME, Mirimanoff RO, Weller M, Cairncross JG, Eisenhauer E, Belanger K, Brandes AA, Allgeier A, Lacombe D, Stupp R (2008). Nomograms for predicting survival of patients with newly diagnosed glioblastoma: prognostic factor analysis of EORTC and NCIC trial 26981-22981/CE.3. Lancet Oncol.

[9] Helseth R, Helseth E, Johannesen TB, Langberg CW, Lote K, Rønning P, Scheie D, Vik A, Meling TR (2010). Overall survival, prognostic factors, and repeated surgery in a consecutive series of 516 patients with glioblastoma multiforme. Acta Neurol Scand.

[10] Lamborn KR, Chang SM, Prados MD (2004). Prognostic factors for survival of patients with glioblastoma: recursive partitioning analysis. Neurooncol.

[11] Walid MS (2008). Prognostic factors for long-term survival after glioblastoma. Perm J.

[12] Lacroix M, Abi-Said D, Fourney DR, Gokaslan ZL, Shi W, Demonte F, Lang FF, McCutcheon IE, Hassenbusch SJ, Holland E (2001). A multivariate analysis of 416 patients with glioblastoma multiforme: prognosis, extent of resection, and survival. J Neurosurg.

[13] Kim YS, Kim SH, Cho J, Kim JW, Chang JH, Kim DS, Lee KS, Suh C-O (2012). MGMT Gene Promoter Methylation as a Potent Prognostic Factor in Glioblastoma Treated With Temozolomide-Based Chemoradiotherapy: A Single-Institution Study. Int J Radiat Oncol Biol Phys.

[14] Malkoun N, Chargari C, Forest F, Fotso M-J, Cartier L, Auberdiac P, Thorin J, Pacaut C, Peoc’h M, Nuti C, Schmitt T, Magné N (2012). Prolonged temozolomide for treatment of glioblastoma: preliminary clinical results and prognostic value of p53 overexpression. J Neuro-Oncol.

[15] Weller M, Felsberg J, Hartmann C, Berger H, Steinbach JP, Schramm J, Westphal M, Schackert G, Simon M, Tonn JC, Heese O, Krex D, Nikkhah G, Pietsch T, Wiestler O, Reifenberger G, von Deimling A, Loeffler M (2009). Molecular predictors of progression-free and overall survival in patients with newly diagnosed glioblastoma: a prospective translational study of the German glioma network. J Clin Oncol.

[16] Onodera T, Goseki N, Kosaki G (1984). prognostic nutritional index in gastrointestinal surgery of malnourished cancer patients. Nihon Geka Gakkai Zasshi.

[17] Ding JD, Yao K, Wang PF, Yan CX (2018). Clinical significance of prognostic nutritional index in patients with glioblastomas. Medicine (Baltimore).

[18] He ZQ, Ke C, Al-Nahari F, Duan H, Guo CC, Wang Y, Zhang XH, Chen YS, Liu ZG, Wang J (2017). Low preoperative prognostic nutritional index predicts poor survival in patients with newly diagnosed high-grade gliomas. J Neuro-Oncol.

[19] Rigamonti A, Imbesi F, Silvani A, Lamperti E, Agostoni E, Porcu L, De Simone I, Torri V, Ciusani E, Bonato C (2019). Prognostic nutritional index as a prognostic marker in glioblastoma: data from a cohort of 282 Italian patients. J Neurol Sci.

[20] Xu W-Z, Li F, Xu Z-K, Chen X, Sun B, Cao J-W, Liu Y-G (2017). Preoperative albumin-to-globulin ratio and prognostic nutrition index predict prognosis for glioblastoma. Onco Targets Ther.

[21] Zhou X-W, Dong H, Yang Y, Luo J-W, Wang X, Liu Y-H, Mao Q (2016). Significance of the prognostic nutritional index in patients with glioblastoma: a retrospective study. Clin Neurol Neurosurg.

[22] Huq S, Khalafallah AM, Botros D, Oliveira LAP, White T, Dux H, Jimenez AE, Mukherjee D (2021). The prognostic impact of nutritional status on postoperative outcomes in glioblastoma. World Neurosurg.

[23] Hayasaka K, Shiono S, Suzuki K, Endoh M, Okada Y (2020). Postoperative prognostic nutritional index as a prognostic factor after non-small cell lung cancer surgery. Gen Thorac Cardiovasc Surg.

[24] Zhang X, Li C, Wen T, Peng W, Yan L, Yang J (2017). Postoperative prognostic nutritional index predicts survival of patients with hepatocellular carcinoma within Milan criteria and Hypersplenism. J Gastrointest Surg.

[25] Murakami Y, Saito H, Kono Y, Shishido Y, Kuroda H, Matsunaga T, Fukumoto Y, Osaki T, Ashida K, Fujiwara Y (2018). Combined analysis of the preoperative and postoperative prognostic nutritional index offers a precise predictor of the prognosis of patients with gastric cancer. Surg Today.

[26] Adejumo OL, Koelling TM, Hummel SL (2015). Nutritional risk index predicts mortality in hospitalized advanced heart failure patients. J Heart Lung Transplant.

[27] Shibutani M, Maeda K, Nagahara H, Ohtani H, Iseki Y, Ikeya T, et al. The prognostic significance of the postoperative prognostic nutritional index in patients with colorectal cancer. BMC Cancer. 2015;15(1):521. 10.1186/s12885-015-1537-x PMC4504172 26177820

[28] Kang M, Chang CT, Sung HH, Jeon HG, Jeong BC, Seo SI, Jeon SS, Choi HY, Lee HM (2017). Prognostic significance of pre- to postoperative dynamics of the prognostic nutritional index for patients with renal cell carcinoma who underwent radical nephrectomy. Ann Surg Oncol.

[29] Borg N, Guilfoyle MR, Greenberg DC, Watts C, Thomson S (2011). Serum albumin and survival in glioblastoma multiforme. J Neuro-Oncol.

[30] Liu W, Qdaisat A, Yeung J, Lopez G, Weinberg J, Zhou S, Cohen L, Bruera E, Yeung SCJ (2019). The association between common clinical characteristics and postoperative morbidity and overall survival in patients with glioblastoma. Oncologist.

[31] Brooks WH, Netsky MG, Normansell DE, Horwitz DA (1972). Depressed cell-mediated immunity in patients with primary intracranial tumors. Characterization of a humoral immunosuppressive factor. J Exp Med.

[32] Kikuchi K, Neuwelt EA (1983). Presence of immunosuppressive factors in brain-tumor cyst fluid. J Neurosurg.

[33] Colotta F, Allavena P, Sica A, Garlanda C, Mantovani A (2009). Cancer-related inflammation, the seventh hallmark of cancer: links to genetic instability. Carcinogenesis.

[34] Kusmartsev S, Gabrilovich DI (2002). Immature myeloid cells and cancer-associated immune suppression. Cancer Immunol Immunother.

[35] Omran ML, Morley JE (2000). Assessment of protein energy malnutrition in older persons, part II: laboratory evaluation. Nutrition.

[36] Seiler WO (2001). Clinical pictures of malnutrition in ill elderly subjects. Nutrition.

[37] Mendez JS, Govindan A, Leong J, Gao F, Huang J, Campian JL (2016). Association between treatment-related lymphopenia and overall survival in elderly patients with newly diagnosed glioblastoma. J Neuro-Oncol.

[38] Nabors LB (2020). Management of Gliomas: Individualized Treatment Options. J Natl Compr Cancer Netw.

[39] Guerrero Orriach JL, Raigon Ponferrada A, Malo Manso A, Herrera Imbroda B, Escalona Belmonte JJ, Ramirez Aliaga M, Ramirez Fernandez A, Diaz Crespo J, Soriano Perez AM, Fontaneda Heredia A, Dominguez Recio ME, Rubio Navarro M, Cruz Mañas J (2020). Anesthesia in combination with Propofol increases disease-free survival in bladder Cancer patients who undergo radical tumor cystectomy as compared to inhalational anesthetics and opiate-based analgesia. Oncology.

[40] Yap A, Lopez-Olivo MA, Dubowitz J, Hiller J, Riedel B (2019). Anesthetic technique and cancer outcomes: a meta-analysis of total intravenous versus volatile anesthesia. Can J Anesth.

