# Supplementary figures and images for: Prognostic significance of the postoperative prognostic nutritional index in patients with glioblastoma: a retrospective study
Source: BMC Cancer. 2021 Aug 21;21:942. doi: 10.1186/s12885-021-08686-8 (PMC8380354; doi:10.1186/s12885-021-08686-8)

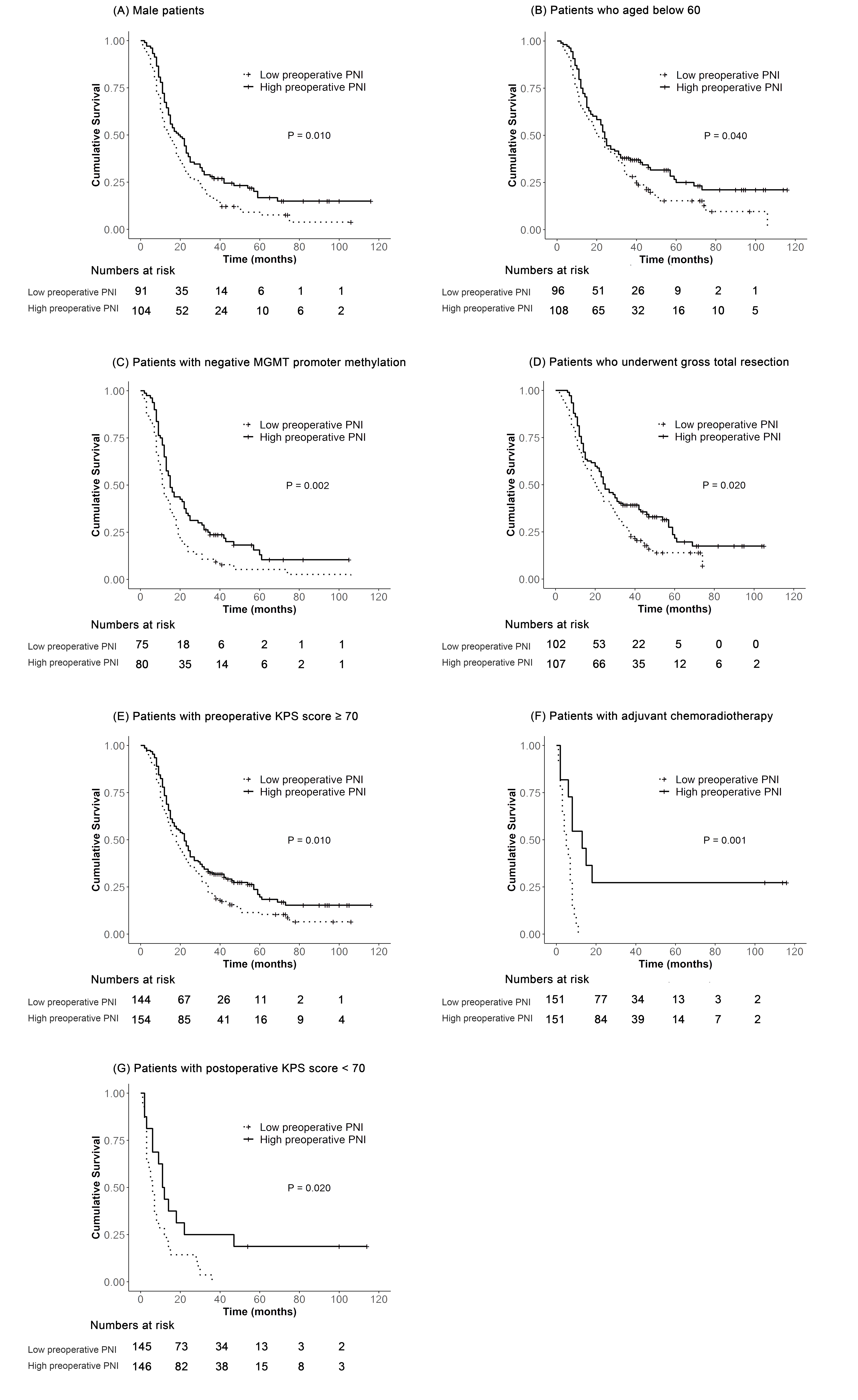

Supplement: Supplementary file 1 — Additional file 1: Supplementary Fig. 1. Kaplan-Meier curves for cumulative survival in (A) male patients, (B) subjects who aged below 60, (C) patients with negative MGMT promoter methylation, (D) patients who underwent gross total resection, (E) patients with preoperative KPS score ≥ 70, (F) patients with adjuvant chemoradiotherapy, and (G) patients with postoperative KPS score < 70. PNI: prognostic nutrition index; MGMT: O6-methylguanine-DNA methyltransferase; KPS: Karnofsky performance status. [file 12885_2021_8686_MOESM1_ESM.tif]
